# Supplementary material for: Aeroponic cultivation of lettuce: Unravelling varietal performance and trait interrelationships for enhanced productivity
Source: PLoS One. 2026 Mar 19;21(3):e0345258. doi: 10.1371/journal.pone.0345258 (PMC13001950; doi:10.1371/journal.pone.0345258)
Supplement: Attachment — Submitted filename: comments1.pdf [file pone.0345258.s001.pdf]

Review Comments to the Author

We sincerely thank the editor and reviewers for overall insightful comments and constructive feedback to improve the MS.

Reviewer #1: The author should provide fully information about cultivars used in this study and also complete background about cultivars

Reply
We sincerely thank the reviewer for this valuable suggestion. We agree that providing clearer background information on the lettuce cultivars improves the scientific context and reproducibility of the study. In response, we have expanded the description of the cultivars used in this experiment.

Also, he must provide detailed information about this cultivars in regular field to compare between yield as this method is effective or not

Reply

We thank the reviewer for this important observation. We agree that comparing aeroponic performance with conventional field cultivation helps in better understanding the effectiveness of aeroponic systems.

However, the present study was specifically designed to evaluate varietal performance and trait interrelationships under controlled aeroponic conditions, and parallel open-field experiments were not conducted during the same season and location.

Also, this investigation must provide us about costs of this method to proof is it effective or not and the increase in yield

Reply

We sincerely thank the reviewer for this insightful comment. We agree that economic considerations are important when assessing the practical effectiveness of aeroponic systems.

The present study was designed as a preliminary investigation focusing on varietal performance, trait interrelationships, and biological productivity of lettuce under aeroponic conditions. Detailed techno-economic and cost–benefit analyses were therefore beyond the scope of this work and will be addressed in future research.

Also, author should explain in details why he choose this lettuce cultivars used in this investigation

Reply

We sincerely thank the reviewer for this constructive comment. We agree that a clear justification for cultivar selection strengthens the scientific rationale of the study.

In response, we have expanded the explanation for cultivar selection to clearly articulate why these specific lettuce cultivars were chosen for evaluation under aeroponic conditions.

Reviewer #2: Add degree symbol in figure 1. Eleventh ....? complete the word. Cite original references for methodology. Check fig 4. SOme of the figures are not clearly visible. Please make them clear. Language should be polished. Introduction part can be reduced.

Response

We thank the reviewer for pointing out this oversight.

Figure 1 has been corrected to include the degree (°) symbol.

The typographical error has been corrected at the indicated location in the manuscript.

Relevant original and standard references (e.g., AOAC, Fisher, Hotelling, Pearson, Lush, and published aeroponic studies) have been cited in the Materials and Methods section.

Figures have been revised with improved resolution

The Introduction has been shortened and refined to improve focus and conciseness.
